# Exploring the crosstalk molecular mechanisms between IgA nephropathy and Sjögren’s syndrome based on comprehensive bioinformatics and immunohistochemical analyses

**DOI:** 10.1007/s10238-024-01420-1

**Published:** 2024-08-13

**Authors:** Peng He, Lei Wei, Ruijing Zhang, Jin Zhao, Yuzhan Zhang, Liuyifei Huang, Xiao Bai, Xiaoxuan Ning, Shiren Sun

**Affiliations:** 1 Department of Nephrology, Xijing Hospital, Fourth Military Medical University, Xi’an, 710032 China; 2 Department of Geriatrics, Xijing Hospital, Fourth Military Medical University, Xi’an, 710032 China

**Keywords:** IgA nephropathy, Sjögren’s syndrome, Autoimmune disease, Bioinformatic analysis, Immunohistochemistry

## Abstract

IgA nephropathy (IgAN) and Sjogren's syndrome (SS) are two autoimmune diseases with undetermined etiology and related to abnormal activation of lymphocytes. This study aims to explore the crucial genes, pathways and immune cells between IgAN and SS. Gene expression profiles of IgAN and SS were obtained from the Gene Expression Omnibus and Nephroseq data. Differentially expressed gene (DEG) and weighted gene co-expression network analyses (WGCNA) were done to identify common genes. Enrichment analysis and protein–protein interaction network were used to explore potential molecular pathways and crosstalk genes between IgAN and SS. The results were further verified by external validation and immunohistochemistry (IHC) analysis. Additionally, immune cell analysis and transcription factor prediction were also conducted. The DEG analysis revealed 28 commonly up-regulated genes, while WGCNA identified 98 interactively positive-correlated module genes between IgAN and SS. The enrichment analysis suggested that these genes were mainly involved in the biological processes of response to virus and antigen processing and presentation. The external validation and IHC analysis identified 5 hub genes (PSMB8, PSMB9, IFI44, ISG15, and CD53). In the immune cell analysis, the effector memory CD8 T and T follicular helper cells were significantly activated, and the corresponding proportions showed positively correlations with the expressions of the 5 hub genes in the two autoimmune diseases. Together, our data identified the crosstalk genes, molecular pathways, and immune cells underlying the IgAN and SS, which provides valuable insights into the intricate mechanisms of these diseases and offers potential intervention targets.

**Supplementary Information:**

The online version contains supplementary material available at 10.1007/s10238-024-01420-1.

## Introduction

Immunoglobulin A (IgA) nephropathy (IgAN) is the most prevalent primary glomerulonephritis worldwide and caused by the deposition of antibodies (IgA) in the glomerular mesangial region [1, 2]. Although the pathogenesis is still not fully understood, there is sufficient evidence to support the IgAN as an autoimmune disease, and a “four-hit hypothesis” (Hit 1, increased production and circulation of galactose-deficient [gd]-IgA1; Hit 2, antiglycan antibodies target gd-IgA1; Hit 3 and Hit 4, formation of immune complexes and mesangial deposition) has been proposed to explain the immune-related mechanism of IgAN [3, 4]. Abnormal activation and inflammatory infiltration of lymphocytes (T and B cells) is one of the prominent pathological features of IgAN [3]. Recently, multiple evidence suggests that intestinal mucosal hyperresponsiveness is related to the activation of B cell subsets through up-regulating the expression of serum B-cell-activating factor (BAFF) and A proliferation-inducing ligand (APRIL), thereby increasing the production of gd-IgA1 [3].

Sjögren’s syndrome (SS) is a chronic autoimmune inflammatory disease characterized by B- and T-cell responses to self-antigens and mostly manifests as sicca syndrome secondary to the lymphocyte infiltration of the epithelial tissues of exocrine glands [5–7]. Besides, extra-glandular manifestations can be seen in approximately three-quarters of patients which occasionally may be life threatening [5–7]. Renal involvement influences approximately 10% patients in the SS, and the most frequent form of nephropathy is tubulointerstitial nephritis, in which infiltration of the kidney by plasma cells is a key feature [8]. Others involve IgAN, membranous nephropathy, membranoproliferative glomerulonephritis secondary to cryoglobulinemia, and focal segmental glomerulosclerosis, etc. [8].

More interesting, according to the relevant reports, the IgAN can not only be secondary to or accompanied by the SS [9, 10], but precede the onset of SS [11]. Although the IgAN and SS share the common pathological feature of kidney impairment associated with the abnormal activation of lymphocytes and can be co-pathogenic, until now, the common molecular landscape and potential mechanisms remain indistinct between the two autoimmune diseases. With the widespread applications of microarray and high-throughput sequencing technology, bioinformatics techniques are frequently done to investigate the crosstalk between miscellaneous diseases. This work attempts to elucidate the common molecular mechanism, action pathways, and characteristic immune cell infiltration from the perspective of potential pathogenic links between IgAN and SS using bioinformatics analysis and immunohistochemistry (IHC) techniques. The goal is to gain a deeper understanding of the pathophysiological processes and to better achieve prediction and personalized medicine for the IgAN and SS.

## Methods

### Access to datasets

We obtained the gene expressions of IgAN and SS, which was downloaded from the GEO database. Datasets are available at https://www.ncbi.nlm.nih.gov/geo/ for more information. Based on the GPL22945 platform, the GSE93798 dataset contains 20 IgAN patients and 22 health controls. These patients had been comprehensively diagnosed by routine kidney biopsy, medical history, and blood examinations. After kidney biopsies, their glomerular compartments were extracted and processed for hybridization on Affymetrix microarrays.

For external verification, we acquired the clinical characteristics and common gene expression data from the Ju CKD Glom (27 IgAN patients and 21 controls) and Ju CKD TubInt (25 IgAN patients and 31 controls) datasets at https://www.nephroseq.org/. Gene expression datasets investigating SS (GSE40611) were based on the GPL570 platform and included 35 parotid gland samples from 17 SS patients and 18 controls. GSE7451 (based on the GPL570 platform), containing 10 IgAN and 10 control salivary gland samples, was used for the external verification of crosstalk genes. The detailed information for the datasets involved in this study is summarized in Supplementary File S1.

### Identification of differentially expressed genes

The original expression matrix was normalized and processed using R (4.3.1) software through Rstudio 2023.06.2 (Windows NT 10.0; Win64; × 64). The “limma” R package was employed to screen the differentially expressed genes (DEGs) from the GSE93798 and GSE40611 datasets with an adjusted *P* value < 0.05 and |log FC|≥ 0.5. The “pheatmap” and “ggplot2” packages were operated to draw a differential gene clustering heatmap and volcano map, respectively.

### Weighted gene co-expression network analysis

The “WGCNA” R package was used to construct the co-expression network. First, the gene expression data were filtered in a “meanFPKM” method. Specifically, we selected the rows from 1 to n orderly using the original data frame and retained the columns with mean FPKM greater than the previously designed thresholds. The retained genes were involved in the weighted gene co-expression network analysis (WGCNA) [12]. Second, hierarchical clustering was done using the standard function “Hculst” to remove the obvious outliers. Third, to ensure the relationship of gene expression consistent with the scale-free network, the function “pickSoftThreshold” was done to confirm the suitable soft threshold. Afterward, the similarity matrices were transformed into an adjacency matrix using the “adjacency” function according to the soft threshold. Fourth, the adjacency matrix was converted into a topological overlap matrix (TOM) for the minimization of spurious associations. Finally, hierarchical clustering and Pearson correlation were employed to detect modules and investigate the correlation between modules and clinical characteristics, respectively. The modules showing powerful correlations with IgAN or SS were chosen, and the corresponding genes were extracted from the selected modules.

### Identification and functional analyses of hub genes

The common genes between IgAN and SS in the DEGs and WGCNA analyses were acquired in the Venn diagrams using the “VennDiagram” R packages and were merged and imported into the STRING database to construct the protein–protein interaction (PPI) network. Terms with adjusted *P* value < 0.05 were considered statistically significant. The network was visualized and analyzed using the Cytoscape (version 3.7.2) software. The cytoHubba analysis in the Cytoscape was employed, and the top 20 genes obtained in the three algorithms, involving maximal clique centrality (MCC), maximum neighborhood component (MNC), and edge percolated component (EPC), were interacted in the Venn diagram to identify the potential hub genes. Gene Ontology (GO) and Kyoto Encyclopedia of Genes and Genomes (KEGG) pathway enrichment analyses were done using the “clusterProfiler” and “ggplot2” packages in R. Statistical significance was set at *P* < 0.05.

### External validation

The expressions of the latent hub genes were externally validated in the Ju CKD Glom, Ju CKD TubInt, and GSE7451 datasets. The Shapiro–Wilks test was done in R to test for the normality of the variables. The comparisons were made using the *t* test or Wilcoxon test in these three datasets. We also conducted the linear correlation analyses between gene expressions and glomerular filtration rates (GFRs, MDRD ml/min/1.73 m^2^) using the Pearson or Spearman methods among the patients with IgAN. A *P* value of < 0.05 was considered significant.

Human renal biopsy samples and IHC analysis.

We collected the human renal biopsy samples from patients with IgAN (*n* = 17, 10 males and 7 females) and renal involvement in SS (*n* = 8, 1 male and 7 females), and para-cancerous kidney tissues (*n* = 3, 2 males and 1 female) were served as normal controls (Supplementary File S2 and S3). These kidney samples were obtained from those treated in Xijing Hospital from January 2017 to December 2019. Histological examination was done in the Department of Pathology of Xijing Hospital, and the corresponding clinical information was collected from the patient's medical records. The study was approved by the Xijing Hospital’s Protection of Human Subjects Committee (Approval number: KY20163162-1), and informed consent was obtained from all patients.

The IHC method was to fix 3–4-μm-thick kidney biopsy sections on glass slides for dewaxing, rehydration, permeation, and sealing. The slide and the primary antibody, PSMB8 (14859-1-AP; dilution 1: 30; ProteinTech, Rosemont, IL, USA) and PSMB9 (14544-1-AP; dilution 1: 75; ProteinTech, Rosemont, IL, USA) polyclonal antibody, were incubated overnight at 4 °C. The slices were then incubated with appropriate biotinylated secondary antibodies and visualized. Non-immunized goat IgG or rabbit IgG was used as a control. Quantification of PSMB8 and PSMB9 after staining was performed by analyzing the % of staining area in at least five randomly selected fields (× 40) of the glomeruli and tubulointerstitium within each section using the ImageJ 1.45 software (National Institutes of Health, Bethesda, USA). Data are expressed as positive stained area vs. total analyzed area. All samples were examined in a blind manner.

### Immune cell and transcription factor analysis

Single-sample GSEA (ssGSEA) was done using the “GSVA” package to analyze the infiltration of 28 immune cells in diseased and normal samples. The Spearman’s rank correlation analysis was employed to test the correlations between the hub genes and the abundances of infiltrating immune cells. Transcription factors (TFs) of the hub genes were predicted in the website of NetworkAnalyst, and a transcriptional regulatory network was yielded (https://www.networkanalyst.ca/).

## Results

### Identification of DEGs in IgAN and SS

**Fig. 1:**
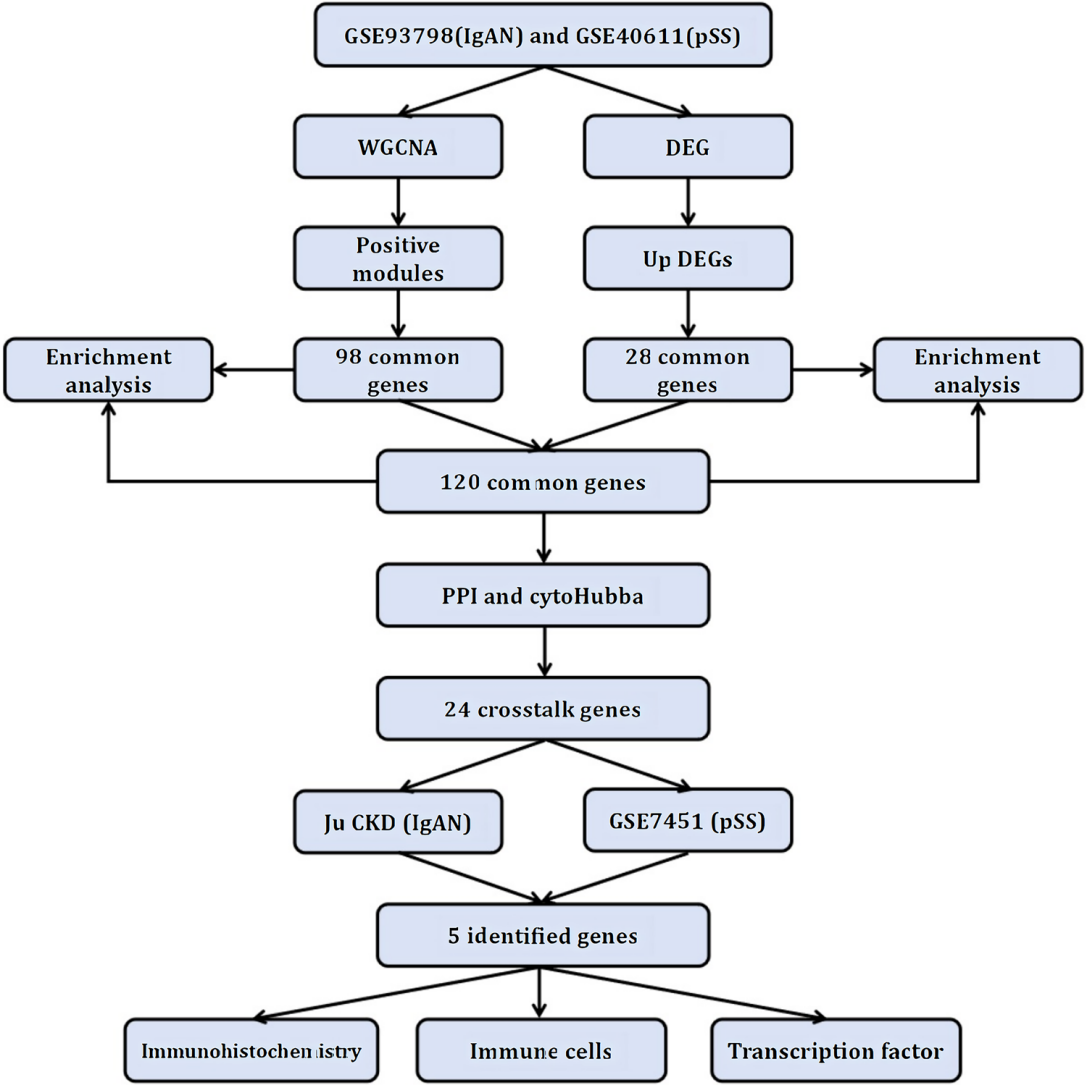
Workflow diagram of the study

**Fig. 2:**
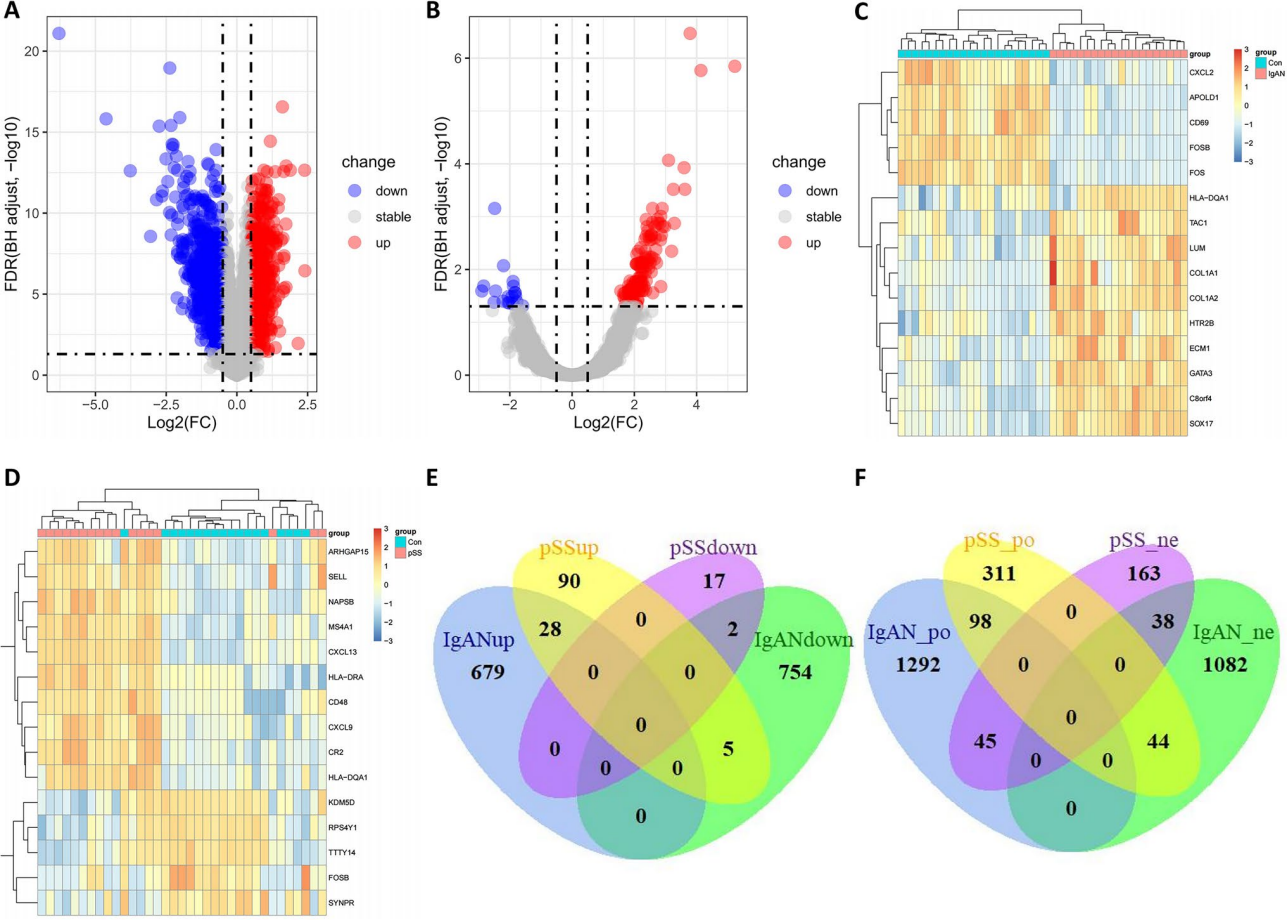
Identification of DEGs in the IgAN and SS. **A** Volcano plot of GSE93798. **B** Volcano plot of GSE40611. **C** Heat map of GSE93798. **D** Heat map of GSE40611. **E** Venn diagram showing the common DEGs between the IgAN and SS. **F** Venn diagram showing the shared genes between the IgAN and SS in the WGCNA modules. DEGs, differentially expressed genes; IgAN, IgA nephropathy; SS, Sjögren’s syndrome; WGCNA, weighted gene co-expression network analysis

The research flowchart is depicted in Fig. 1. DEG analyses were performed using GSE93798 and GSE40611 datasets. In GSE93798, we obtained 1468 DEGs, involving 707 up-regulated and 761 down-regulated genes, while in GSE40611, 142 DEGs were found, containing 123 up-regulated and 19 down-regulated genes, respectively. These results are presented in the volcano plots (Fig. 2A and B). We also labeled the top 10 up-regulated and top 5 down-regulated genes in the heatmaps (Fig. 2C and D). Subsequently, 28 commonly up-regulated and 2 commonly down-regulated DEGs between IgAN and SS were identified using the Venn diagram (Fig. 2E).

To further uncover the latent biological processes and pathways, GO and KEGG analyses were done using the commonly up-regulated DEGs. The results indicated that these DEGs were principally enriched in lymphocyte-mediated immunity, MHC class II protein complex assembly, antigen processing and presentation, virus and staphylococcus aureus infections, and autoimmune diseases (Supplementary File S4).

### WGCNA in IgAN and SS

**Fig. 3:**
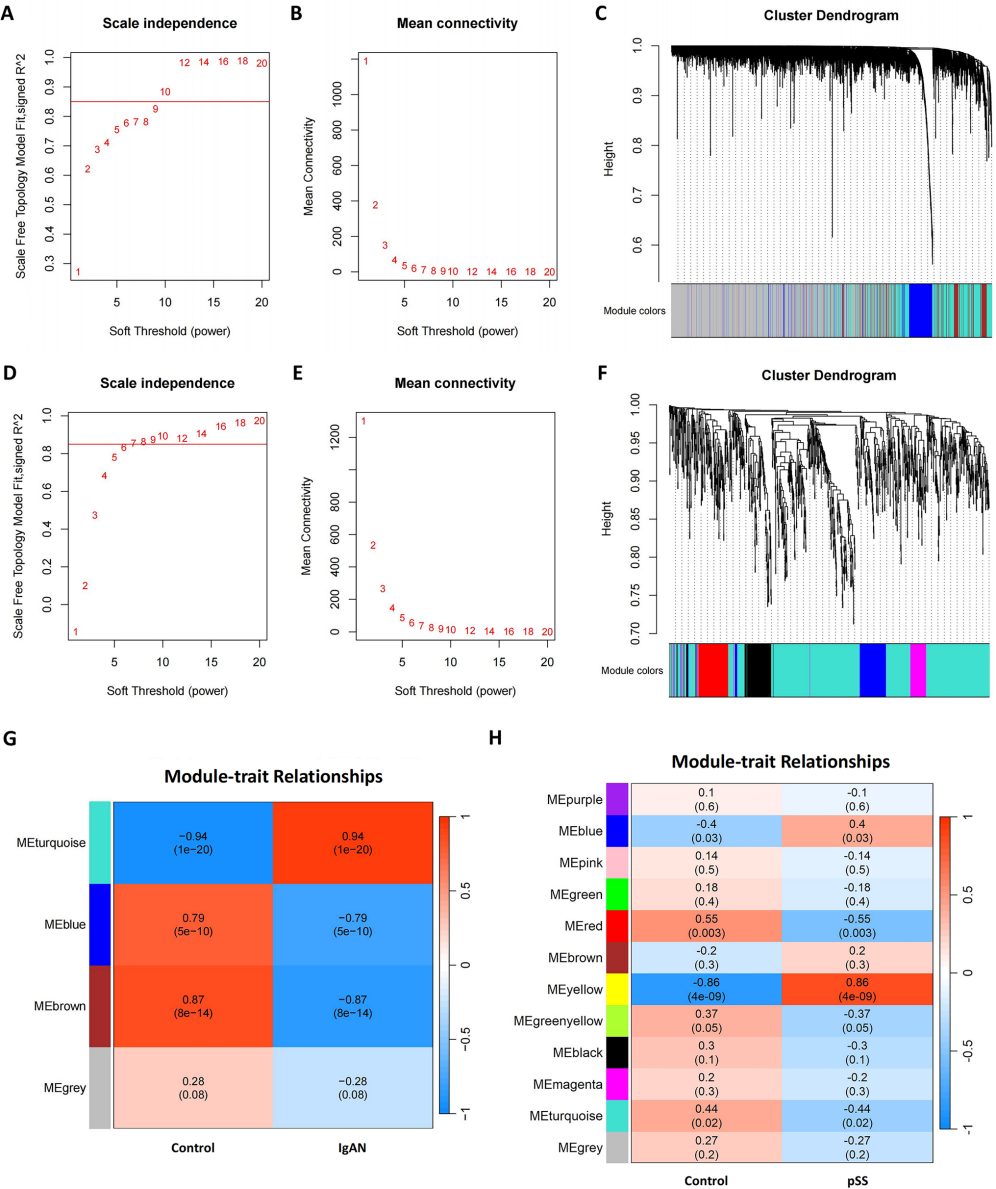
WGCNA in the IgAN and SS. **A** Scale independent plot in the GSE93798. **B** Mean connectivity plot in the GSE93798. **C** Cluster dendrogram in the GSE93798. **D** Scale independent plot in the GSE40611. **E** Mean connectivity plot in the GSE40611. **F** Cluster dendrogram in the GSE40611. **G** Heat map of the module-trait relationships in the GSE93798. **H** Heat map of the module-trait relationships in the GSE40611

A total of 5753 and 4665 genes were preserved with a meanFPKM value of 7 and 9 in the GSE93798 and GSE40611 datasets, respectively, and the function “Hculst” locked 42 and 28 samples, respectively, for the subsequent processing. The “pickSoftThreshold” function yielded a proper soft threshold value of 10 in IgAN and 7 in SS, respectively (Fig. 3A, B, D, and E). In the GSE93798 and GSE40611 datasets, four and twelve modules with diverse colors were screened out in the WGCNA, respectively (Fig. 3C and F).

The relationship between these modules and corresponding disease was computed using the Spearman correlation coefficient heatmap. In the GSE93798, the “MEturquoise” module, including 1435 genes, showed a significantly positive correlation with IgAN (*R* = 0.94, *P* = 1e−20), while the “MEblue” and “MEbrown” modules were negatively correlated with IgAN (*R* = − 0.79, *P* = 5e−10; *R* = − 0.87, *P* = 8e−14) and comprised a total of 1164 genes (Fig. 3G). Similarly, in the GSE40611, the “MEyellow” module, containing 453 genes, showed a high positive correlation with SS (*R* = 0.86, *P* = 4e−09), while the “MEred” module had a negative correlation with SS (*r* = − 0.55, *P* = 0.003), comprising 246 genes (Fig. 3H).

Afterward, 98 interactively positive-correlated and 38 interactively negative-correlated module genes between IgAN and SS were identified though the Venn diagram (Fig. 2F). The enrichment analyses suggested that the commonly positive-correlated module genes were primarily enriched in the antigen processing and presentation, response to virus, staphylococcus aureus infections, and autoimmune diseases, which were consistent with the results of DEG analysis (Supplementary File S5).

### Identification of hub genes between IgAN and SS

**Fig. 4:**
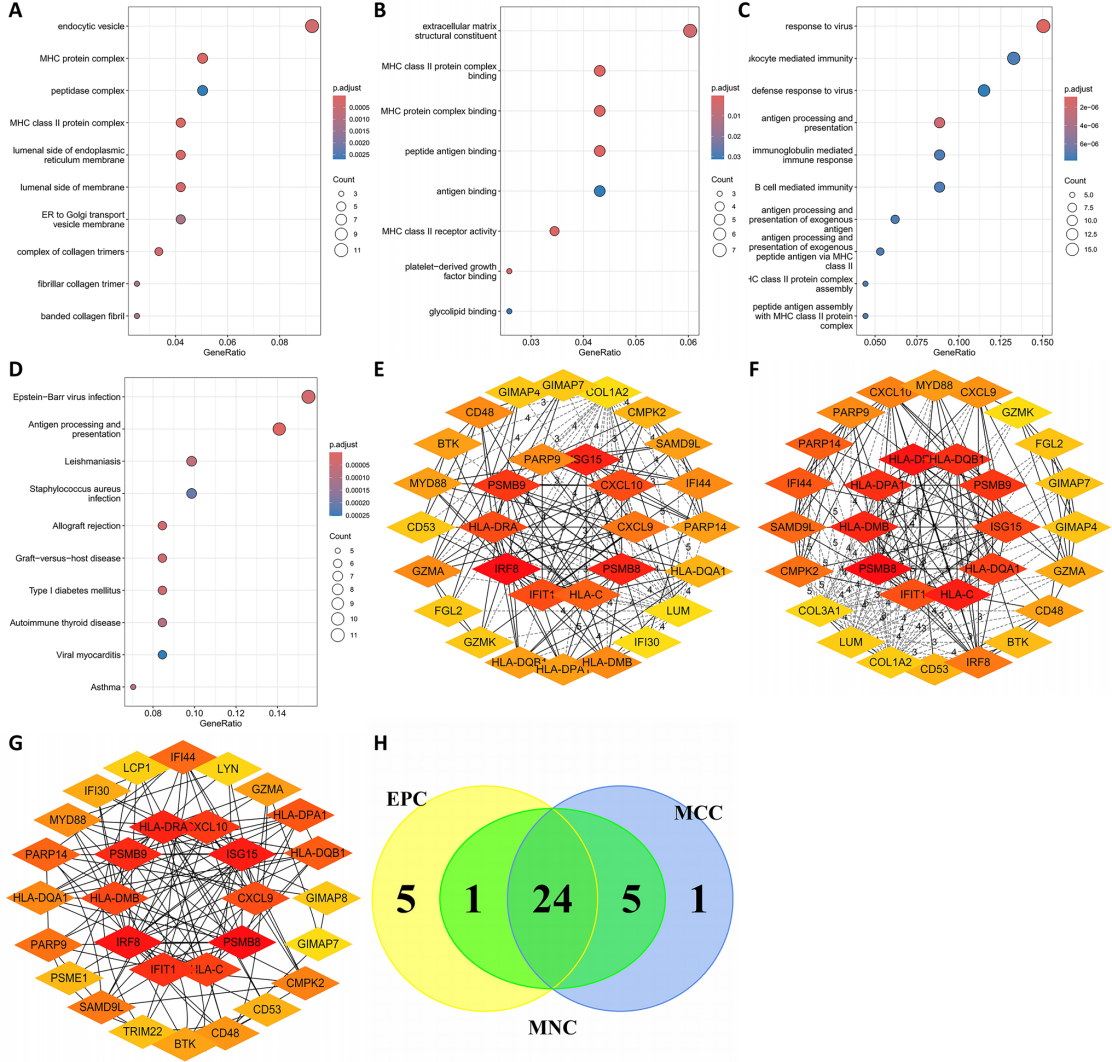
GO, KEGG, and PPI analyses. The **A** cellular component, **B** molecular function, **C** biological process, and **D** KEGG enrichment analyses of the 120 crosstalk genes. The PPI networks between the top 30 genes in the **E** MCC, **F** MNC, and **G** EPC analyses. **H** Venn diagram showing the 24 potential hub genes between the IgAN and SS. GO, gene ontology; KEGG, Kyoto Encyclopedia of Genes and Genomes; PPI, protein–protein interaction; MCC, maximal clique centrality; MNC, maximum neighborhood component; EPC, edge percolated component

The 28 commonly up-regulated DEGs and 98 interactively positive-correlated module genes between IgAN and SS were merged, and 120 genes were acquired for the subsequent research. As shown in Fig. 4A–D, the results of GO and KEGG enrichment analyses highlighted the crucial roles of antigen processing and presentation and response to virus in the biological processes and pathways in both autoimmune diseases. Subsequently, we employed PPI analysis through the STRING site and three algorithms of the plug-in cytoHubba (MCC, MNC, and EPC) in the Cytoscape to investigate the core pathogenic genes. The interaction networks display the top 30 genes that obtained using the MCC, MNC, and EPC algorithms, respectively (Fig. 4D–F). By intersecting the Venn diagrams (Fig. 4G), 24 shared genes were identified as the candidate hub genes, including PSMB8, PSMB9, IFI44, ISG15, CD53, HLA-DQA1, HLA-DQB1, HLA-C, HLA-DMB, HLA-DRA, HLA-DPA1, GZMA, GIMAP7, CXCL9, CXCL10, CMPK2, SAMD9L, PARP14, PARP9, IFIT1, BTK, IRF8, MYD88, and CD48.

### Validation of hub gene expressions between IgAN and SS

**Fig. 5:**
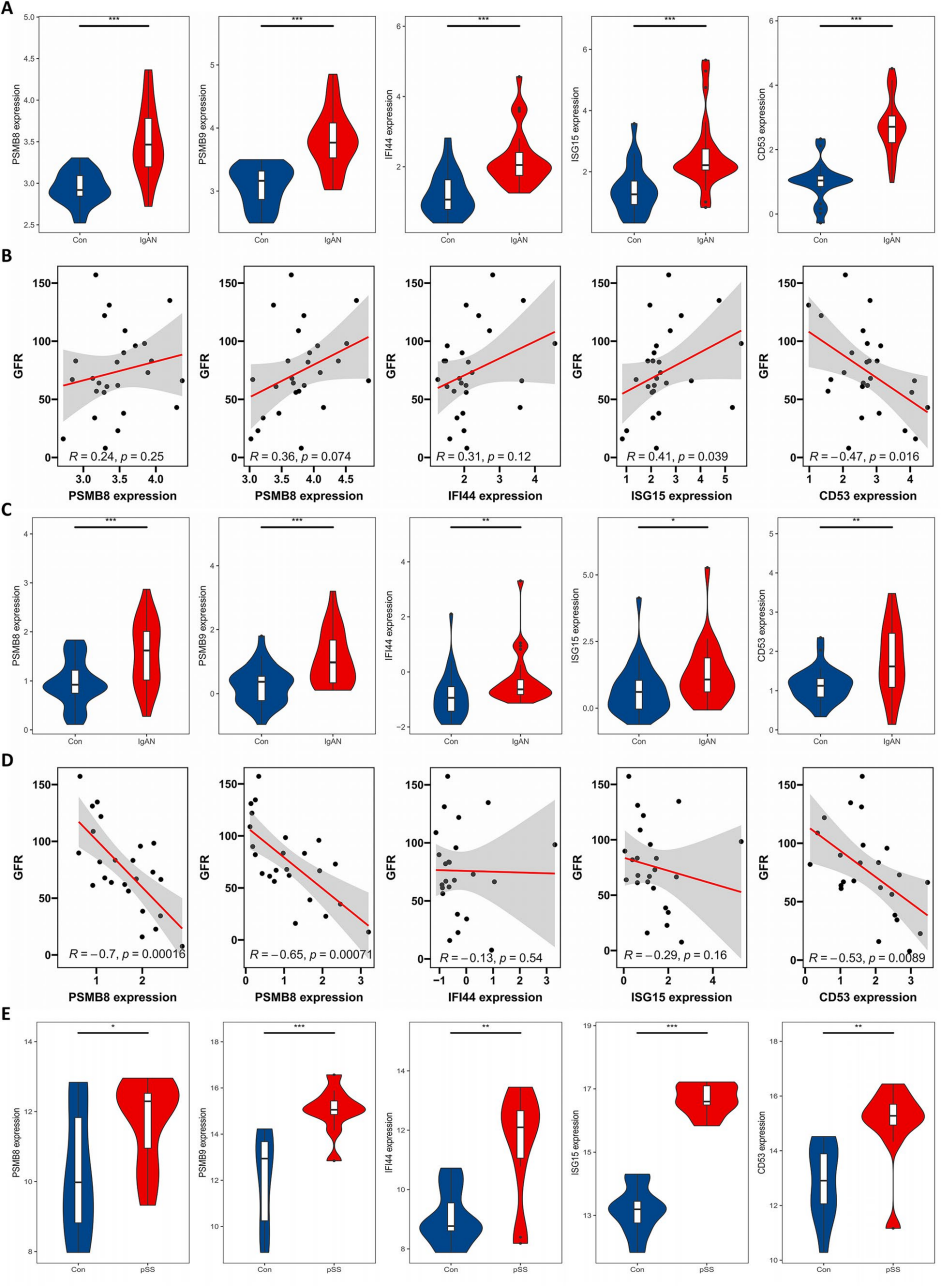
External validation of the hub gene expressions and correlation analyses. The expressions of the five hub genes in the **A** Ju CKD Glom, **B** Ju CKD TubInt, and **C** GSE7451 datasets. Correlation analyses between the expression levels of the five hub genes and the GFR (ml/min/1.73 m.^2^) using the **D** Ju CKD Glom and **E** Ju CKD TubInt datasets. *P* value < 0.05 was considered statistically significant. *P < 0.05, **P < 0.01, ***P < 0.001

The expression levels of the 24 genes were externally validated in the IgAN dataset Ju CKD and SS dataset GSE7451. The results indicated that the expressions of PSMB8, PSMB9, IFI44, ISG15, and CD53 in the IgAN glomerular samples were obviously higher than those in the samples of healthy controls, respectively (Fig. 5A). More interestingly, the expressions of ISG15 in the glomerular samples exhibited a remarkably positive correlation, while the CD53 showed a negative correlation with the GFR values of corresponding IgAN patients, respectively (*R* = 0.41, *P* = 0.039; *R* = − 0.47, *P* = 0.016; Fig. 5B). Similar analyses were done in the Ju CKD TubInt. The results demonstrated that the PSMB8, PSMB9, IFI44, ISG15, and CD53 expressions were higher in the IgAN tubulointerstitial samples than those in the controls, respectively, and the PSMB8, PSMB9, and CD53 expressions showed negative correlations with the GFR values (*R* = − 0.7, *P* = 0.00016; *R* = − 0.65, *P* = 0.00071; *R* = − 0.53, *P* = 0.0089), respectively, in the IgAN tubulointerstitial samples (Fig. 5C and D).

On the other hand, in the GSE7451, the expression levels of PSMB8, PSMB9, IFI44, ISG15, and CD53 were significantly increased in the salivary gland samples of SS than those in the samples of healthy controls, respectively (Fig. 5E). Consequently, according to these comparative results, PSMB8, PSMB9, IFI44, ISG15, and CD53 were selected as the hub genes between IgAN and SS for the subsequent analyses.

**Fig. 6:**
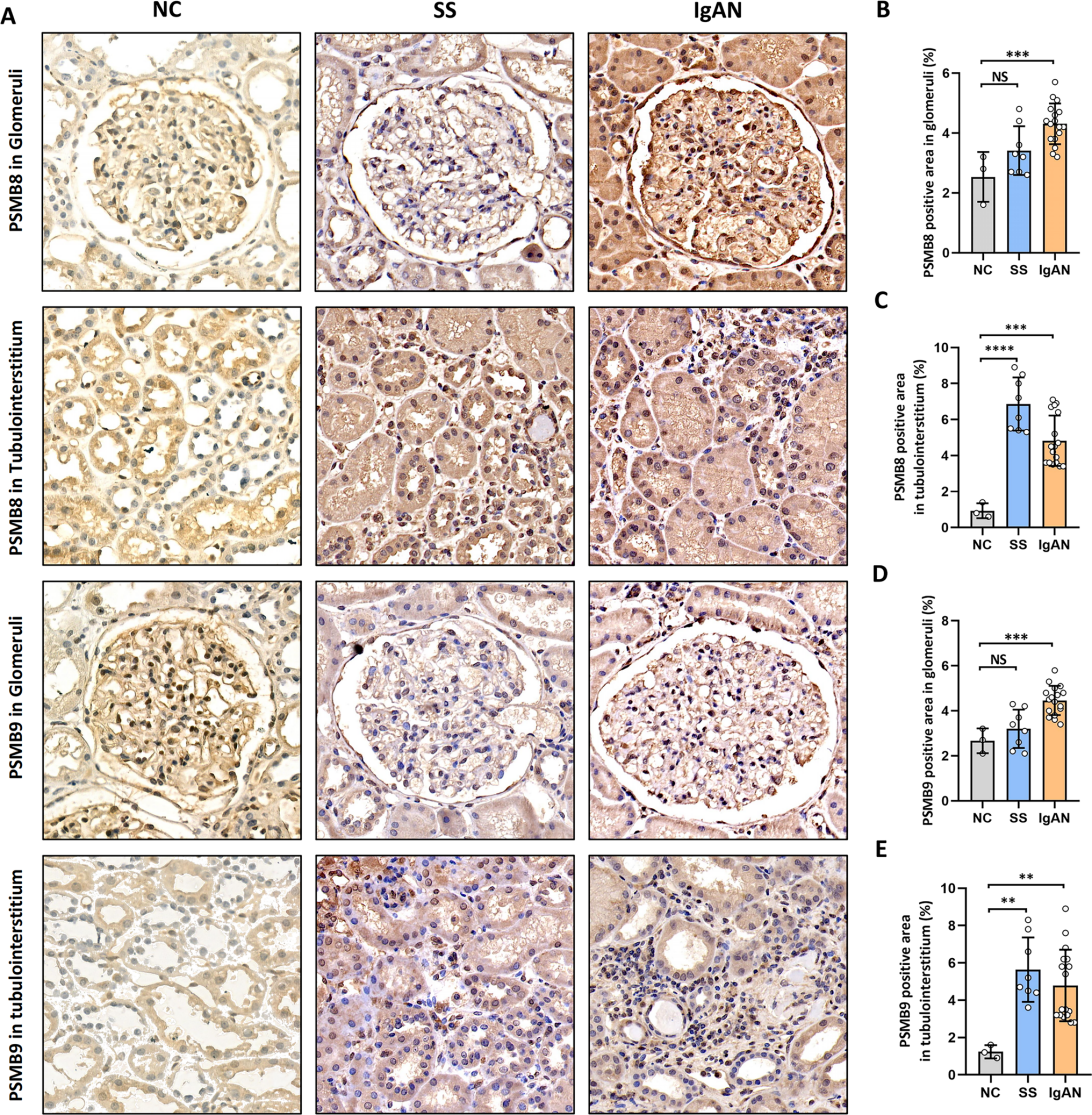
External validation of the PSMB8 and PSMB9 expressions using the IHC methods. **A** Photomicrographs of PSMB8 and PSMB9 in the glomeruli and tubulointerstitium of IgAN, SS, and controls. Quantifications of the PSMB8 expressions in the **B** glomeruli and **C** tubulointerstitium of IgAN and controls. Quantifications of the PSMB9 expressions in the **D** glomeruli and **E** tubulointerstitium of IgAN and controls. *P* value < 0.05 was considered statistically significant. *P < 0.05, **P < 0.01, ***P < 0.001, ****P < 0.0001

In the IHC analyses, the expressions of PSMB8 and PSMB9 were significantly increased in the glomeruli and tubulointerstitium of IgAN than those in the controls, respectively (Fig. 6A–C). As for the patients with renal involvement in SS, the expression levels of PSMB8 and PSMB9 were mainly elevated in the tubulointerstitium (Fig. 6A, D, and E). Furthermore, the PSMB8 and PSMB9 levels in the tubulointerstitium were negatively correlated with the eGFR values of patients with renal involvement in SS and IgAN, respectively, and the proteinuria levels of patients with IgAN, respectively (Supplementary File S6). As for the Oxford classification of IgAN (M [Mesangial hypercellularity], E [Endocapillary proliferation], S [Segmental sclerosis], T [Tubular atrophy and interstitial fibrosis], and C [Crescentic] scores), the PSMB8 and PSMB9 levels in the tubulointerstitium were markedly increased in the T1/2 groups than those in the T0 groups, respectively (Supplementary File S7). Additionally, we also observed that the PSMB9 levels in the glomeruli were elevated in the M1, S1, and C1 groups, respectively, though the lack of statistical significance in our data (Supplementary File S7).

### Immune cell infiltration and correlation analyses

**Fig. 7:**
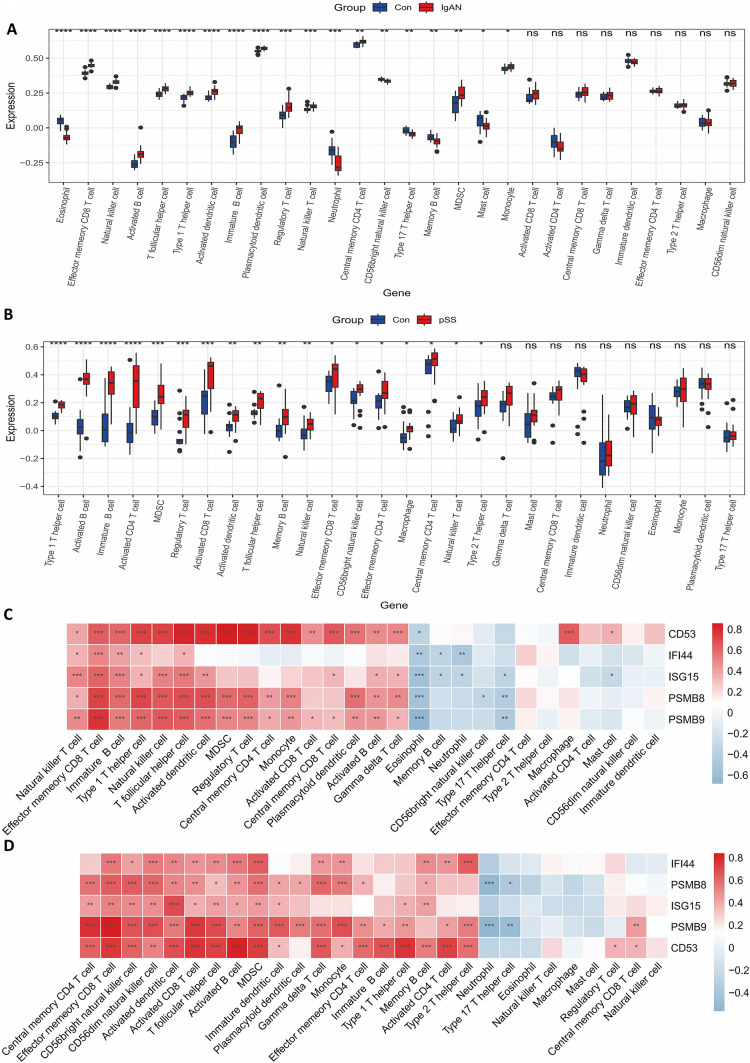
Immune infiltration analyses associated with the IgAN and SS. **A** The box plot showing the distribution of 28 immune cells in the GSE93798. **B** The box plot showing the distribution of 28 immune cells in the GSE40611. **C** Correlation analysis between immune cells and the hub genes in the GSE93798. **D** Correlation analysis between immune cells and the hub genes in the GSE40611. *P* value < 0.05 was considered statistically significant. *P < 0.05, **P < 0.01, ***P < 0.001, ****P < 0.001

The immune cell infiltration analyses were done in different samples. The distributions of 28 types of immune cells in the GSE93798 and GSE40611 samples were identified and are presented in the box plots in Fig. 7A and B. We observed that various types of immune cells were significantly activated both in the IgAN and SS samples, including effector memory (EM) CD8 T cell, T follicular helper (Tfh) cell, type 1 T helper cell, regulatory T cell, natural killer T cell, central memory CD4 T cell, immature B cell, activated B cell, natural killer cell, activated dendritic cell, and myeloid-derived suppressor cell (MDSC).

The correlations between the infiltrated immune cells and the expressions of hub genes are depicted in Fig. 7C, D, and Supplementary File S5. The EM CD8 T cell, Tfh cell, central memory CD4 T cell, activated B cell, activated dendritic cell, and MDSC were positively correlated to the expressions of PSMB8 and PSMB9, respectively. Most of the activated immune cells mentioned above, except for nature killer cell and nature killer T cell, exhibited remarkably positive correlations with the expressions of CD53 in IgAN and SS, respectively. More interestingly, we noticed that the EM CD8 T cell and Tfh cell showed significantly positive correlations with the expressions of all the five hub genes, respectively, and activated B cell and activated dendritic cell were positively related to the PSMB8, PSMB9, ISG15, and CD53, respectively, in the two diseases. Therefore, these data indicate that the EM CD8 T cell, Tfh cell, activated B cell, and activated dendritic cell may exert dominant effects in the pathogenesis of IgAN and SS.

### TF prediction and validation

According to the NetworkAnalyst, we have identified twenty-eight TFs that may participate in the transcriptional regulatory of our five crosstalk genes, including STAT1, FOXC1, USF1, CREB1, YY1, POU2F2, GATA2, GATA3, TP53, MAX, etc. The transcriptional regulatory network is presented in the Supplementary File S6A. As shown in the Supplementary File S5B, the expressions of STAT1 were remarkably elevated in SS (GSE7451) and IgAN tubulointerstitium (Ju CKD TubInt) than healthy controls. We also observed that the expression of STAT1 showed a significantly negative correlation with GFR values in the tubulointerstitium of IgAN (*R* = − 0.44; *P* = 0.033; Supplementary File S5C). Together, these results demonstrate a possible role of STAT1 in the transcriptional regulatory of ISG15 and CD53 both in the IgAN and SS.

## Discussion

In this research, we aimed to explore the crosstalk target genes, related pathways, and possible TFs between IgAN and SS through the integrative bioinformatic analyses of transcriptomes and IHC technique. First of all, we computed and combined the shared genes in the WGCNA modules and the common DEGs of IgAN and SS. The GO and KEGG enrichment analyses indicated that these genes were mainly involved in the biological processes of response to virus and antigen processing and presentation. Afterward, in the PPI and cytoHubba analyses, we acquired 24 crosstalk genes between IgAN and SS, and then identified 5 hub genes (PSMB8, PSMB9, IFI44, ISG15, and CD53) in the validation datasets. The immune cell infiltration demonstrated that the EM CD8 T and Tfh cells were obviously activated in the IgAN and SS, and the corresponding proportions exhibited positively correlations with the expressions of the 5 hub genes. Additionally, we predicted and verified the STAT1 as the possible TF of the ISG15 and CD53 in the two autoimmune diseases.

As we known, the infections activate the mucosal immune system, which has been recognized to contribute to the onset of IgAN, causing it to produce excess IgA, leading to the deposition [1, 2, 13]. In the renal specimens of IgAN patients, the antigens of Human Cytomegalovirus, Adeno and Herpes simplex virus, Epstein-Barr virus, and even Staphylococcus have been detected, accompanied by the IgA deposits [13, 14]. Likewise, numerous epidemiological and experimental investigations provide evidence of an association between prior viral infection and subsequent development of SS, including Epstein-Barr virus, Cytomegalovirus, hepatitis C virus, etc. [7, 15, 16]. The abnormal interference of exogenetic virus or endogenetic viral elements leads to the activation of mucosal epithelial cells and immune systems with the auto-antibody secretion [5]. These auto-antibodies are aggregated into immune complexes that induce the production of interferon (IFN) *α*, resulting in a cycle of immune-system activation that leads to tissue damage [5]. In our research, the shared genes between IgAN and SS were dramatically enriched in the biological processes of response to virus and the pathways of Epstein-Barr virus, Influenza A, and Human T-cell leukemia virus 1 infection, Staphylococcus aureus infection, and intestinal immune network for IgA production. These data indicate that the invasion of specific pathogens and mucosal immune response may exert non-negligible roles in the pathogenesis of the two diseases, and corresponding clinical interventions may be beneficial to the disease control.

The PSMB8 and PSMB9 genes code for low molecular weight protein 7 (LMP7) and LMP2, respectively, involved in the proteasome switch and antigen processing to generate major histocompatibility complex class I binding peptides and then contribute to the activation of lymphocyte in the IgAN [17]. In a genome-wide association research, rs9357155 at the PSMB8/9 locus was related to an increased risk of IgAN [18]. In the SS, PSMB8 has been reported to be up-regulated and hypo-methylated in the salivary glands [19, 20], and PSMB9 appears to be a good predictor for the classification with a fold change of 2.81 and an adjusted *P* value of 9.29e−07 [21]. Consistent with these findings, we recognized and verified the PSMB8 and PSMB9 as the hub genes, and the enrichment analyses revealed the key role of antigen processing and presentation in the pathogenesis of IgAN and SS. Therefore, we speculate that the abnormal antigen processing and presentation may be one of the pathogenic factors in the two autoimmune diseases, and several core molecules in this pathway have the potential to serve as the indicators for the diagnosis and disease assessment.

Another three hub genes between the IgAN and SS that we screened were CD53, IFI44, and ISG15. The CD53 encodes a tetraspanin, which is known to be expressed by different immune cells, and exerts important and non-redundant roles in the immune cell adhesion and migration, and regulation of immune cell signaling [22]. A bioinformatics study showed that the CD53 could be a meaningful classifier for the SS with a fold change of 3.11 and an adjusted *P* of 2.52E−06 [21]. Our data also demonstrated that the expressions of CD53 in the glomeruli and tubulointerstitium of IgAN were negatively correlated with the GFR, respectively. It would be of great benefit to explore the pathophysiological functions of CD53 in the antigen-presenting cells of IgAN and SS and to further extend findings to primary cells and in vivo models. The IFN systems are unusually activated in the peripheral blood and impaired tissue of SS and IgAN patients resulting in the activation of autoimmune response and tissue damage which was mentioned above. IFI44 is a type I IFN signature gene, which may participate in the pathogenesis of autoimmune diseases [23]. As one of the earliest IFN-stimulated genes (ISGs), ISG15 is abnormally up- or down-regulated in multiple types of cancer and infectious diseases [24].

In the immune cell infiltration analyses, we observed that the EM CD8 + T cell and Tfh cell showed significant positive correlations with the five hub genes, respectively. Tfh cell is a CD4 + T cell subset that promotes B cell maturation and differentiation, antibody production, and formation of germinal center in lymphoid follicles, and plays critical roles in the pathogenesis of many autoimmune diseases, such as systemic lupus erythematosus, rheumatoid arthritis, SS, systemic sclerosis, etc. [25]. Significantly increased proportion of activated cTfh cells and cTfh/follicular regulatory T cell ratio are found in SS patients [26]. Higher frequency of PD-1^+^ICOS^+^ Tfh cells is positively correlated with higher level of autoantibodies, ESR, IgG, cytokines, and disease activity as well [27]. In the IgAN, recent studies showed that Tfh cells in tertiary lymphoid structure contributed to the renal fibrosis by IL-21 and the renal progression by activating B cells cell–cell interactions [28, 29]. Additionally, several studies indicated that the EM CD8 + T cell subset, in the SS, for example, had high cytolytic activity and was more efficient in migrating to inflamed peripheral tissues during the effector phase of immune response than other EM subsets and took part in pathogens clearance [30, 31]. More valuable studies are needed to uncover the pathophysiological role of EM CD8 + T cells in the various phases of the immune response in the IgAN. Taken together, these data highlight the critical impact of specific T cell subsets in the pathogenesis of the IgAN and SS.

The study has several strengths. We used the systematic bioinformatics analysis as a new approach to explore the relationship between the IgAN and SS. Reasonable internal and external validation, and IHC methods further improved the robustness of the conclusions. The application of immune infiltration analysis made it possible to explore the common immune microenvironment of the two diseases. There are also some limitations in our study. In the selected cohorts, some clinical information was not considered for analyses, such as age, sex, ethnicity, medication, comorbidities of patients, etc. The influence of these characteristics on the results cannot be ignored. Therefore, large-scale observational studies should be carried out to verify the value of these core molecules and exclude the heterogeneity caused by other possible factors. Additionally, further experimental study is needed to confirm our findings in this work, which provides novel insights for further studies on molecular biological mechanisms of IgAN and SS.

In conclusion, our work depicted the common molecule, pathway, and immune cell features of the IgAN and SS using the bioinformatics analysis and experimental validation. A better understanding of the pathogenesis of each disease plays a critical role in identifying new targets for early decision making and intervention from the perspective of prediction and personalized medicine.

## Supplementary Information

Below is the link to the electronic supplementary material.

Supplementary file1 (DOCX 2314 KB)

## Data Availability

The raw data supporting the conclusions of this article will be made available by the authors without undue reservation. The data in the current study are available from the corresponding author upon reasonable request. And the public data can be acquired below: https://www.ncbi.nlm.nih.gov/geo/query/acc.cgi?acc=GSE93798, https://www.ncbi.nlm.nih.gov/geo/query/acc.cgi=GSE40611, https://www.ncbi.nlm.nih.gov/geo%20/query/acc.cgi?acc=GSE7451, and https://www.nephroseq.org/.
