# Supplementary material for: Exploring the crosstalk molecular mechanisms between IgA nephropathy and Sjögren’s syndrome based on comprehensive bioinformatics and immunohistochemical analyses
Source: Clin Exp Med. 2024 Aug 13;24(1):188. doi: 10.1007/s10238-024-01420-1 (PMC11322200; doi:10.1007/s10238-024-01420-1)
Supplement: Supplementary file 1 — Supplementary file1 (DOCX 2314 KB) [file 10238_2024_1420_MOESM1_ESM.docx]

**Exploring the** **crosstalk molecular mechanisms between IgA nephropathy and** **Sjögren’s syndrome based on comprehensive** **bioinformatics and** **immunohistochemical analyses**

Peng He^1†^, Lei Wei^1†^, Ruijing Zhang^1†^, Jin Zhao^1^, Yuzhan Zhang^1^, Liuyifei Huang^1^, Xiao Bai^1^, Xiaoxuan Ning^2‡^ , and Shiren Sun^1‡^

^1^ Department of Nephrology, Xijing Hospital, Fourth Military Medical University, Xi’an 710032, China

^2^ Department of Geriatrics, Xijing Hospital, Fourth Military Medical University, Xi’an 710032, China

^†^ These authors have contributed equally to this work

^‡^ Correspondence should be addressed to ningxx01@fmmu.edu.cn (X.N.) and [sunshiren@medmail.com.cn](mailto:sunshiren@medmail.com.cn) (S.S.)

Supplementary File S1. General information of the included datasets

| **No.** | **Data number** | **Platform** | **Samples** | **Source types** | **Disease** |
| --- | --- | --- | --- | --- | --- |
| 1 | GSE93798 | GPL22945 | 20 IgAN/22 HC | Glomeruli | IgAN |
| 2 | GSE40611 | GPL570 | 17 SS/18 HC | Parotid | SS |
| 3 | Ju CKD Glom | NA | 27 IgAN/21 HC | Glomeruli | IgAN |
| 4 | Ju CKD TubInt | NA | 25 IgAN/31 HC | Tubulointerstitium | IgAN |
| 5 | GSE7451 | GPL570 | 10 SS/10 HC | Saliva | SS |

IgAN, IgA nephropathy; SS, Sjögren’s syndrome; HC, healthy control; CKD, chronic kidney disease.

Supplementary File S2. Clinical information of the included patients with SS and NCs

| Patient No. | Gender | Age, years | Disease | Race | eGFR, ml/min/1.73m^2^ |
| --- | --- | --- | --- | --- | --- |
| 1 | Male | 52 | NC | Yellow | 107.9 |
| 2 | Female | 46 | NC | Yellow | 109.2 |
| 3 | Male | 59 | NC | Yellow | 104.8 |
| 4 | Female | 25 | SS | Yellow | 92 |
| 5 | Female | 34 | SS | Yellow | 123.7 |
| 6 | Male | 44 | SS | Yellow | 36.2 |
| 7 | Female | 33 | SS | Yellow | 43.7 |
| 8 | Female | 61 | SS | Yellow | 94.7 |
| 9 | Male | 59 | SS | Yellow | 83.2 |
| 10 | Male | 47 | SS | Yellow | 96 |
| 11 | Female | 35 | SS | Yellow | 72 |

NC, normal control; eGFR, estimated glomerular filtration rate.

Supplementary File S3. Clinical information of the included patients with IgAN

| Patient No. | Gender | Age  (years) | Disease | Race | eGFR  (ml/min/1.73m^2^) | Proteinuria  (g/24h) | M | E | S | T | C |
| --- | --- | --- | --- | --- | --- | --- | --- | --- | --- | --- | --- |
| 1 | male | 51 | IgAN | Yellow | 23.5 | 2.0 | 1 | 0 | 1 | 2 | 0 |
| 2 | male | 26 | IgAN | Yellow | 75.8 | 0.5 | 0 | 0 | 0 | 0 | 0 |
| 3 | male | 26 | IgAN | Yellow | 19.9 | 6.0 | 1 | 0 | 1 | 2 | 0 |
| 4 | female | 52 | IgAN | Yellow | 17.3 | 5.8 | 1 | 0 | 1 | 2 | 1 |
| 5 | female | 27 | IgAN | Yellow | 71 | 3.5 | 1 | 1 | 1 | 0 | 1 |
| 6 | female | 42 | IgAN | Yellow | 91 | 0.5 | 1 | 0 | 1 | 0 | 0 |
| 7 | male | 27 | IgAN | Yellow | 76.1 | 0.9 | 1 | 0 | 1 | 0 | 0 |
| 8 | female | 41 | IgAN | Yellow | 96.3 | 1.3 | 0 | 0 | 0 | 0 | 0 |
| 9 | male | 30 | IgAN | Yellow | 17.9 | 5.6 | 1 | 0 | 1 | 2 | 1 |
| 10 | male | 28 | IgAN | Yellow | 90.2 | 6.8 | 0 | 0 | 0 | 0 | 0 |
| 11 | male | 33 | IgAN | Yellow | 47.5 | 5.4 | 1 | 1 | 1 | 0 | 0 |
| 12 | male | 37 | IgAN | Yellow | 84.3 | 0.5 | 1 | 1 | 1 | 0 | 0 |
| 13 | female | 80 | IgAN | Yellow | 67.4 | 0.2 | 1 | 1 | 1 | 0 | 0 |
| 14 | male | 39 | IgAN | Yellow | 80.4 | 0.8 | 0 | 0 | 1 | 0 | 1 |
| 15 | female | 49 | IgAN | Yellow | 65.1 | 0.8 | 0 | 0 | 0 | 0 | 1 |
| 16 | female | 31 | IgAN | Yellow | 42.4 | 3.3 | 0 | 1 | 1 | 1 | 0 |
| 17 | male | 29 | IgAN | Yellow | 61.6 | 0.4 | 0 | 0 | 0 | 0 | 0 |

M, Mesangial hypercellularity; S, Segmental sclerosis; E, Endocapillary proliferation; T, Tubular atrophy and interstitial fibrosis; C, Crescentic.


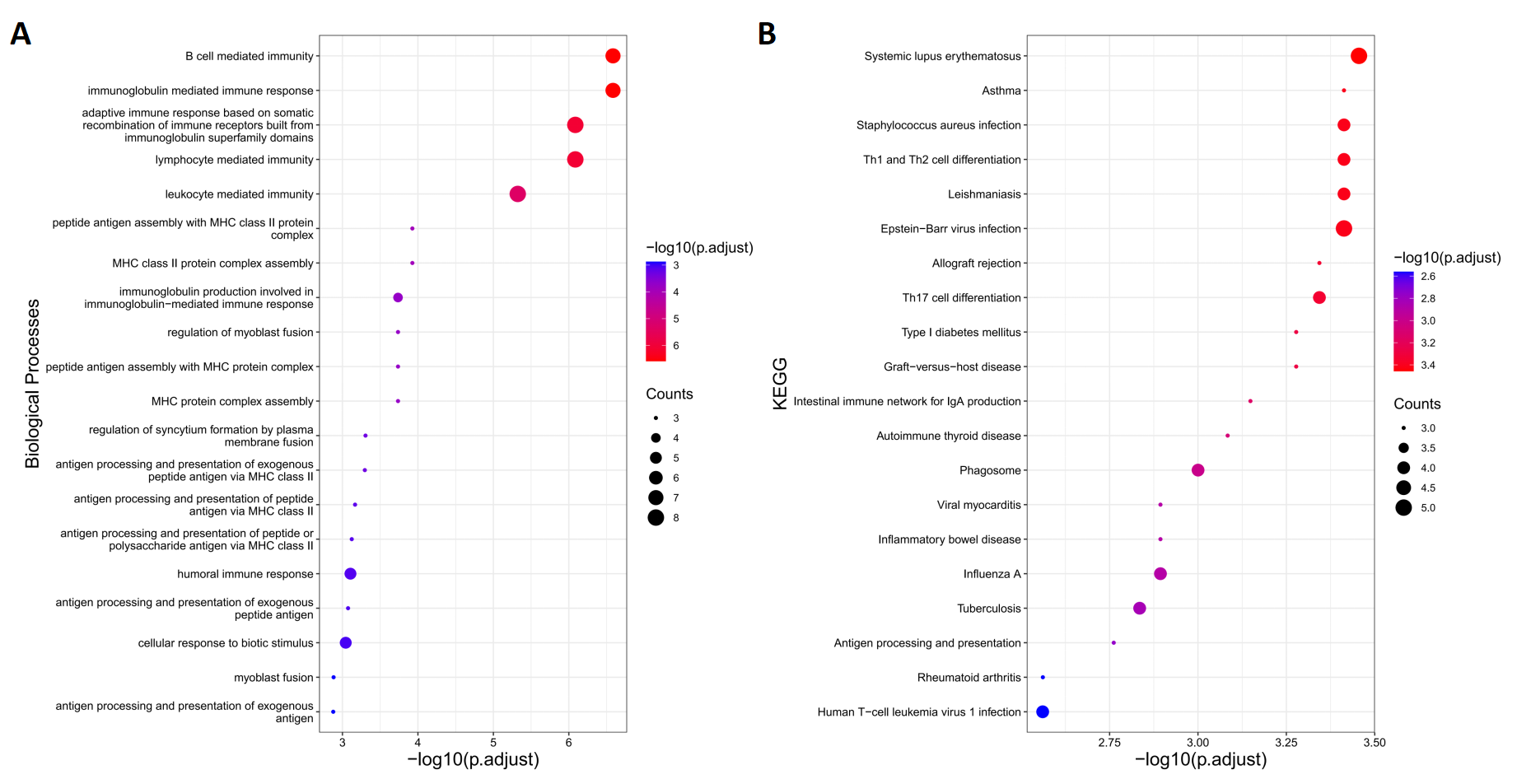


Supplementary File S4. Biological process (**A**) and pathway analyses (**B**) using the commonly up-regulated DEGs between IgAN and SS. DEG, differentially expressed genes.


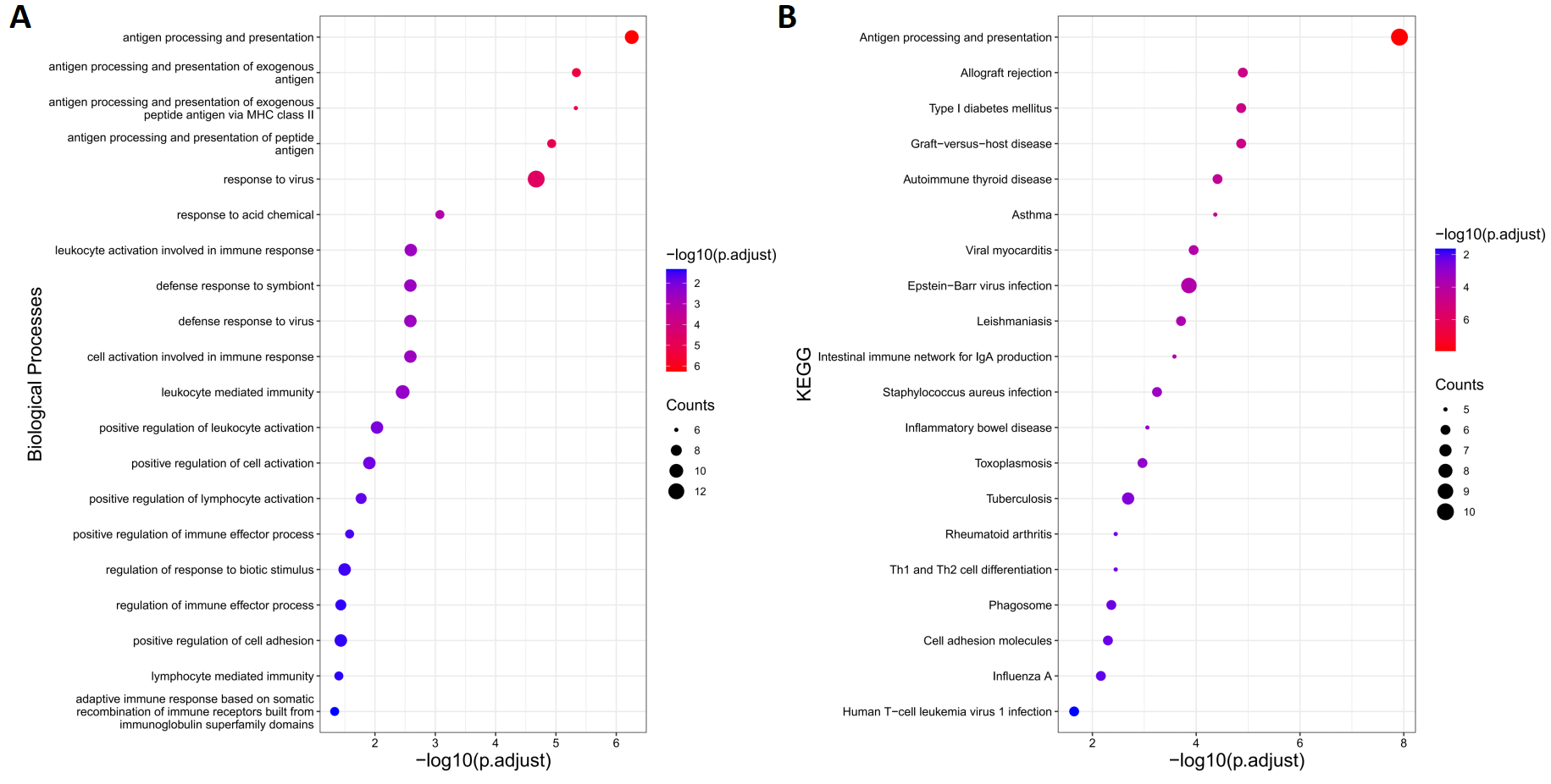


Supplementary File S5. Biological process (**A**) and pathway analyses (**B**) using the commonly positive-correlated module genes between IgAN and SS.


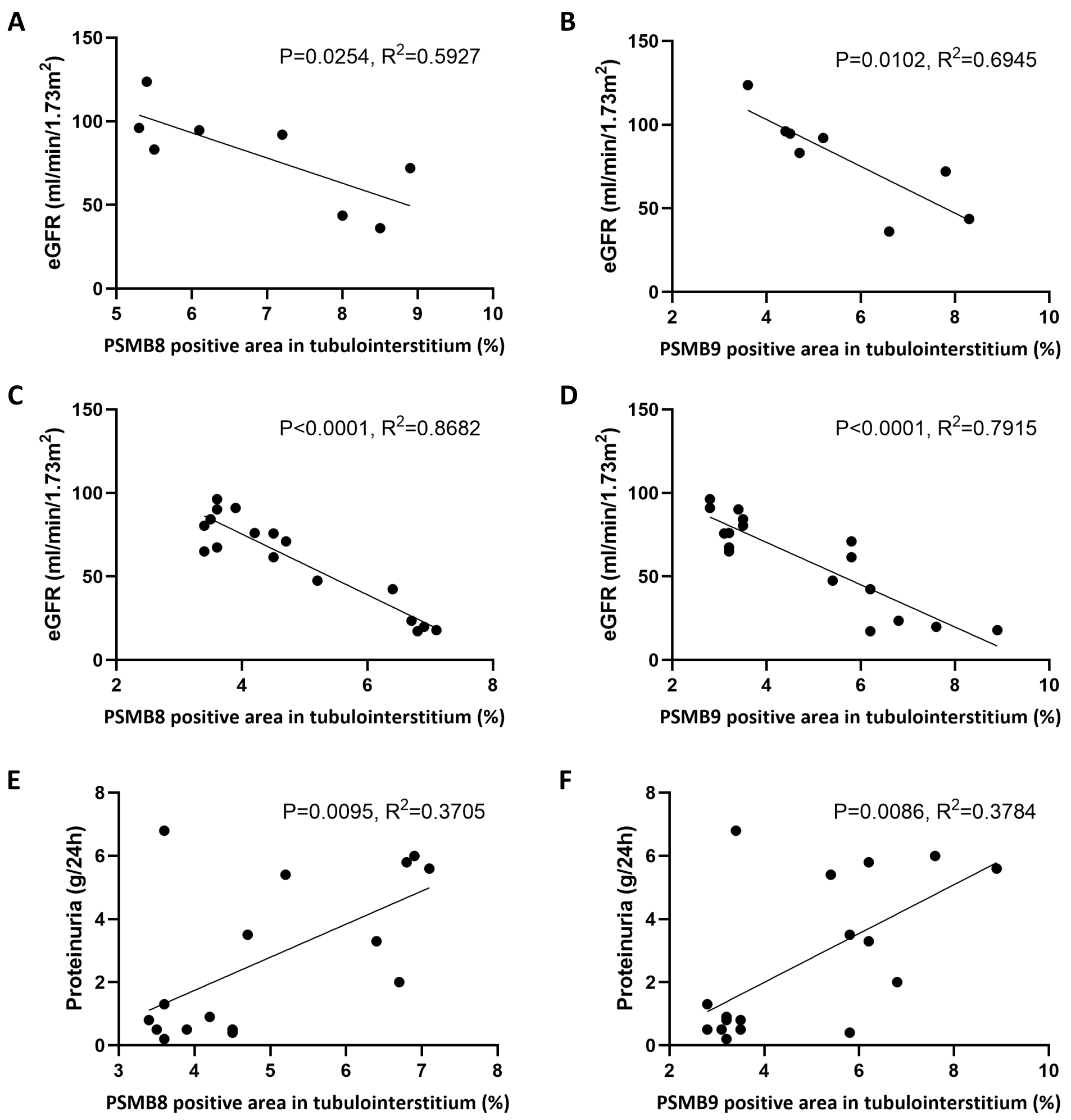


Supplementary File S6. Correlation analysis between PSMB8 positive areas in tubulointerstitium and eGFR values of patients with SS (**A**). Correlation analysis between PSMB9 positive areas in tubulointerstitium and eGFR values of patients with SS (**B**). Correlation analysis between PSMB8 positive areas in tubulointerstitium and eGFR values of patients with IgAN (**C**). Correlation analysis between PSMB9 positive areas in tubulointerstitium and eGFR values of patients with IgAN (**D**). Correlation analysis between PSMB8 positive areas in tubulointerstitium and proteinuria values of patients with IgAN (**E**). Correlation analysis between PSMB9 positive areas in tubulointerstitium and proteinuria values of patients with IgAN (**F**).


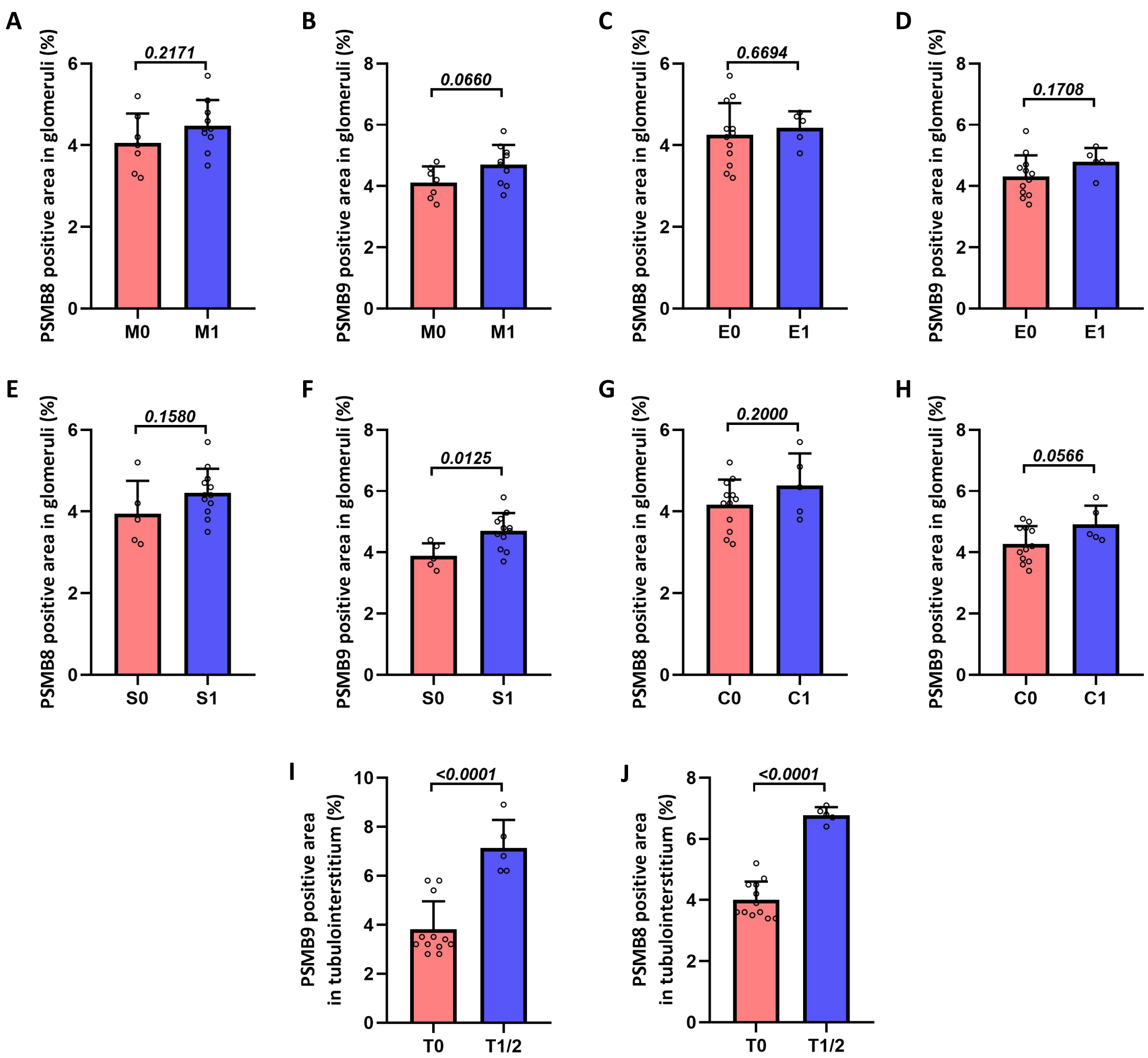


Supplementary File S7. Differences of PSMB8 or PSMB9 positive areas in the Oxford classification of IgAN.

Supplementary File S8. Summary of the immune cell analyses in IgAN and SS


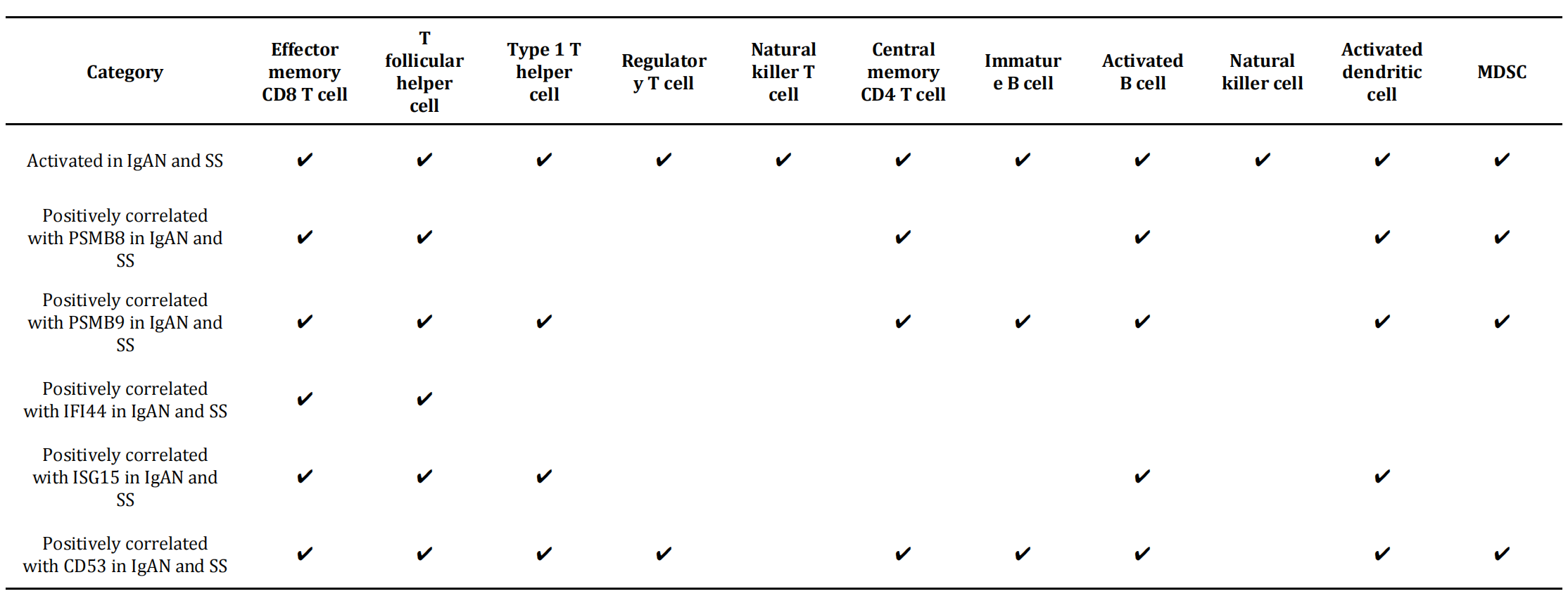


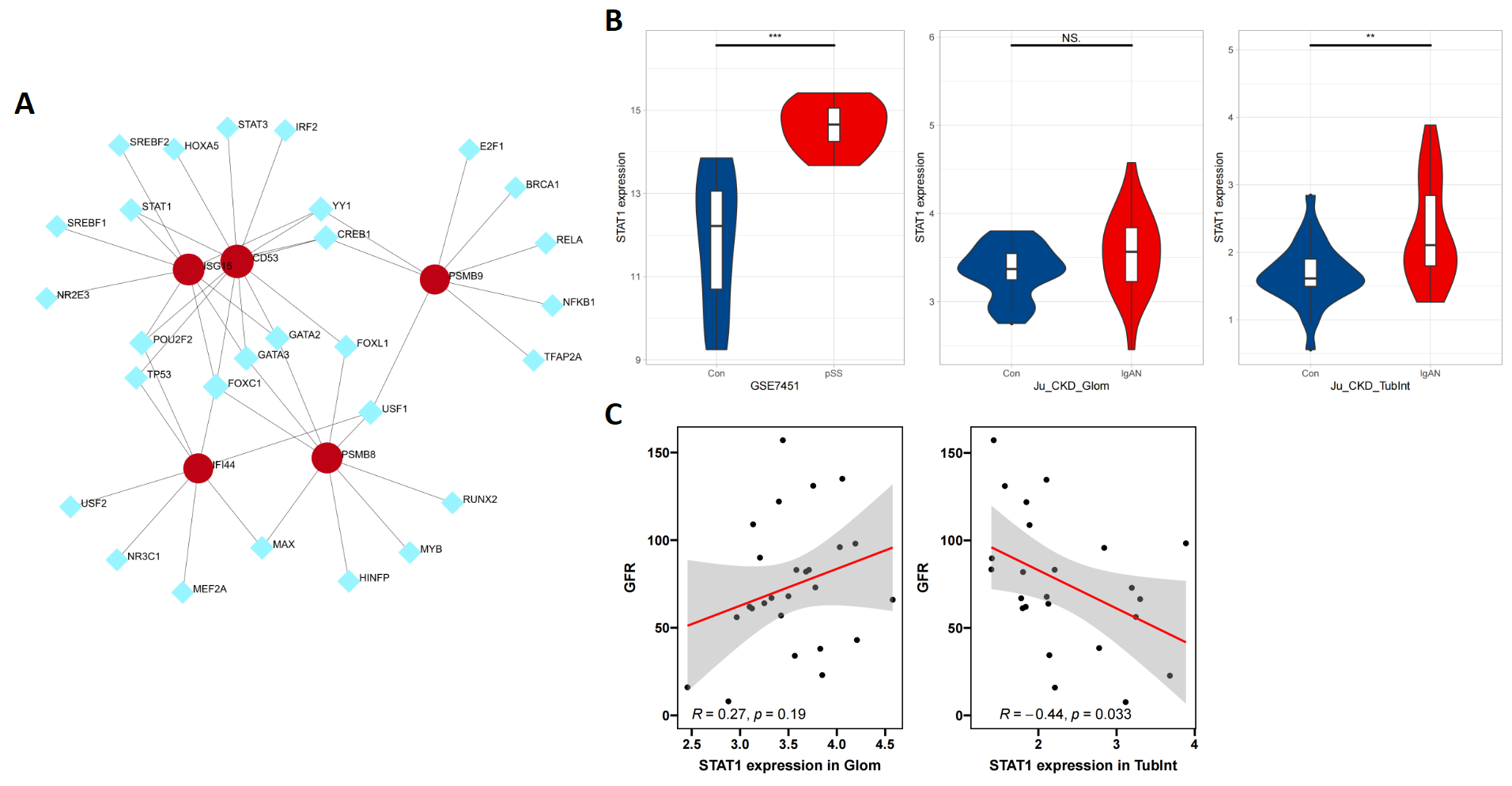


Supplementary File S9. TF prediction and validation. (A) The transcriptional regulatory network of the five hub genes. (B) The expressions of the STAT1 in the Ju CKD Glom, Ju CKD TubInt, and GSE7451 datasets. (C) The correlation analyses of the STAT1 in the Ju CKD Glom and TubInt datasets. P value < 0.05 was considered statistically significant. *P < 0.05, **P < 0.01, ***P < 0.001.
